# Proposed Diagnostic Criteria for the *DSM-5* of Nonsuicidal Self-Injury in Female Adolescents: Diagnostic and Clinical Correlates

**DOI:** 10.1155/2013/159208

**Published:** 2013-07-31

**Authors:** Tina In-Albon, Claudia Ruf, Marc Schmid

**Affiliations:** ^1^Clinical Child and Adolescent Psychology, University of Koblenz-Landau, Ostbahnstrasse 12, 76829 Landau, Germany; ^2^Department of Clinical Psychology and Psychotherapy, Institute of Psychology, University of Basel, 4055 Basel, Switzerland; ^3^Department of Child and Adolescent Psychiatry, University of Basel, 4056 Basel, Switzerland

## Abstract

Nonsuicidal self-injury (NSSI) is included as conditions for further study in the *DSM-5*. Therefore, it is necessary to investigate the proposed diagnostic criteria and the diagnostic and clinical correlates for the validity of a diagnostic entity. The authors investigated the characteristics of NSSI disorder and the proposed diagnostic criteria. A sample of 73 female inpatient adolescents and 37 nonclinical adolescents (aged 13 to 19 years) was recruited. Patients were classified into 4 groups (adolescents with NSSI disorder, adolescents with NSSI without impairment/distress, clinical controls without NSSI, and nonclinical controls). Adolescents were compared on self-reported psychopathology and diagnostic cooccurrences. Results indicate that adolescents with NSSI disorder have a higher level of impairment than adolescents with other mental disorders without NSSI. Most common comorbid diagnoses were major depression, social phobia, and PTSD. There was some overlap of adolescents with NSSI disorder and suicidal behaviour and borderline personality disorder, but there were also important differences. Results further suggest that the proposed *DSM-5* diagnostic criteria for NSSI are useful and necessary. In conclusion, NSSI is a highly impairing disorder characterized by high comorbidity with various disorders, providing further evidence that NSSI should be a distinct diagnostic entity.

## 1. Introduction

Given the prevalence of nonsuicidal self-injury (NSSI) [1, 2], its related problems [3, 4], and the findings that it is often present in individuals who are not diagnosed with borderline personality disorder (BPD) [5], NSSI should be considered a distinct diagnostic category. Currently, NSSI is not in the classification system of the fourth edition of the *Diagnostic and Statistical Manual of Mental Disorders* (*DSM-IV)* or the *International Classification of Diseases*, tenth revision (*ICD-10)* as a distinct entity, but it does exist as a symptom of BPD. So far, several attempts have been undertaken to include an NSSI disorder in the DSM [6, 7], the most recent for the upcoming fifth edition, the *DSM-5* [8]. For the *DSM-5* NSSI is included as conditions for further study, indicating that criteria sets will need further research before it will be an official diagnosis [9]. The most important justification is clearly the clinical benefit that a distinct diagnosis for NSSI leads to a better understanding, management, and specific treatment. Previously, Muehlenkamp [6] proposed more generally that repetitive NSSI should be established as a diagnostic entity to improve research on this behavior. More recently, Wilkinson and Goodyer [10] proposed in addition to the clinical benefit several positive consequences if NSSI were to be classified as a diagnosis in its own right, such as improving communication between professionals and patients and increasing research into the nature, course, and outcome of NSSI. In addition a diagnosis is also the base to provide financing from health insurances. Currently many patients with NSSI are officially diagnosed with their comorbid diagnoses or with BPD even without fulfilling all required criteria, although, NSSI is their main problem and therefore the main goal of psychotherapy should focus on NSSI. However, without an official diagnosis there is a discrepancy and intransparency between communication to the patient and the health insurance companies. As there is now a definition for NSSI and suggested diagnostic criteria for the *DSM-5*, it is necessary to test these criteria and to have diagnostic and clinical correlates. 

In a recent adolescent community study [11] the prevalence rate of NSSI using the proposed criteria for *DSM-5* was 6.7%. However, regarding criterion D it was not assessed whether adolescents self-injured during states of psychosis nor whether they engaged in NSSI when not intoxicated [11]. Data from clinical samples are to our knowledge not available. 

One important aspect of a new distinct entity that is also relevant for diagnostic validity is its delimitation in respect to other disorders [12]. Regarding NSSI, a clear differentiation from BPD is needed. Self-injurious behavior is one of nine symptoms of BPD in the *DSM-IV-TR. *However, although NSSI and BPD can cooccur, they also occur independently. Even early reports warned against subsuming NSSI under a specific personality disorder. Several studies indicated that only about 50% of those who engage in NSSI suffer from BPD [5, 13, 14]. These studies had the limitation that at the time of their investigations, diagnostic criteria for NSSI were not yet available, and thus they used different definitions of NSSI that are not comparable, such as that NSSI has to be engaged in repeatedly (on 5 or more days in the last year). In a retrospective chart review, Selby et al. [15] compared treatment-seeking adult outpatients who engaged in NSSI with a group with BPD as well as a comparison group with various Axis I diagnoses. The NSSI and BPD groups had similar levels of impairment and psychopathology. The NSSI group was characterized by higher depressive symptoms, anxiety, and suicidality than the clinical comparison group. However, most of the NSSI group did not exhibit subthreshold BPD symptoms. As the data were obtained from the charts, no information was available about frequency and motivation for NSSI. Nevertheless, results indicated that NSSI has the potential to be a separate diagnostic entity. 

Another important yet difficult distinction has to be made between NSSI and attempted suicide. Three key differences are noteworthy. First, most people engaging in NSSI have, per definition, no intent to die during the self-injuring act. Second, methods and injuries of  NSSI are often less severe and usually the damage is not life threatening. Third, NSSI and suicide differ in the frequency of the act, as NSSI often occurs daily [16, 17]. Nevertheless, it is important to highlight that longitudinal studies show that NSSI is a significant predictor for suicidal behavior and most people engaging in NSSI report suicidal ideation [18–20]. 

The issue of an unclear definition of NSSI also applies for studies investigating methods of NSSI and diagnostic and clinical correlates. Nock et al. [13] and Hintikka et al. [21] investigated diagnostic correlates in adolescents with NSSI. The most common Axis I disorders in adolescents with NSSI were major depressive disorder, conduct disorder, and PTSD [13, 21]. In the study by Nock et al. [13], 67.3% of the sample met criteria for a *DSM-IV* personality disorder, of which BPD was most common (51.7%). Regarding methods and characteristics of NSSI, Nixon et al. [22] investigated 42 hospitalized adolescents with repetitive NSSI. All endorsed cutting and/or scratching. More than 80% reported almost daily urges to self-injure, and more than 60% reported at least once-a-week acts of self-injury. Seventy-four percent of the adolescents reported having attempted suicide at least once in the past 6 months. Axis II disorders or symptoms of BPD were not assessed in the Nixon et al. [22] study, nor impairment or distress due to NSSI. Clinical correlates indicate that patients with NSSI have difficulties in emotion regulation [23] and, as found in studies of diagnostic correlates, elevated depression as well as externalizing and borderline symptomatology [15, 24, 25]. 

As yet, there have been precious few empirical studies investigating diagnostic and clinical correlates using the proposed *DSM-5* criteria for NSSI and therefore little data support the validity of the criteria. Thus, our aim was threefold: first, to investigate the proposed diagnostic criteria for NSSI for the *DSM-5* using a clinical interview with inpatient female adolescents; second, to examine the diagnostic and clinical correlates of adolescents with NSSI disorder; and third, to compare adolescents with NSSI disorder with adolescents with no mental disorders, adolescents with mental disorders without NSSI, and subgroups of adolescents with NSSI such as adolescents with NSSI who did not report impairment or distress. We hypothesized that adolescents with NSSI disorder can be differentiated from other clinical and nonclinical groups. That adolescents with NSSI disorder would be more likely to have a history of suicide attempts, would have more comorbid diagnoses and score higher on self-reported psychopathology, especially borderline symptoms, and would have difficulties in emotion regulation and be more impaired in global functioning compared with the other groups.

## 2. Method

### 2.1. Participants

Participants were 110 female adolescents, aged 13–18 years, recruited from different inpatient psychiatric units in Switzerland and Germany. Participants included 41 adolescents who fulfilled the proposed *DSM-5* criteria for NSSI disorder, 12 adolescents with NSSI but denied being impaired or distressed due to NSSI, 20 adolescents with a *DSM-IV* diagnosis other than NSSI, and 37 nonclinical adolescents who had no current or past experience of mental disorder. Adolescents with repetitive NSSI but who denied being impaired or distressed due to NSSI were the only subgroup in the NSSI group that could be used for further analyses. These adolescents indicated in the diagnostic interview repetitive NSSI but denied the questions on impairment and distress in different settings such as family, school, or leisure. In addition they denied questions such as if the patient has to hide the wounds and scars in daily life, if the patient thinks about possible long term consequences of the behavior, and how difficult it would be to stop from one day to the other with NSSI. Demographic and psychosocial characteristics of adolescents with NSSI disorder, adolescents with NSSI without impairment/distress, clinical controls, and nonclinical controls are reported in Table 1. The samples were different with respect to age (*F* = 6.14, *p* < .01). Post hoc analysis indicated that this effect was mainly due to the younger age of the nonclinical adolescents group. 

### 2.2. Procedure

All participants and their parents were informed about the study and gave their written consent in accordance with the Declaration of Helsinki. The local ethics committee approved the study. 

### 2.3. Measures: Assessment of Axis I and Axis II Diagnoses

To examine the participants' current or past *DSM-IV-TR* diagnoses for Axis I disorders, we conducted a structured interview with each adolescent. The *Diagnostic Interview for Mental Disorders in Children and Adolescents* [45, Kinder-DIPS] assesses the most frequent mental disorders in childhood and adolescence (all anxiety disorders, depression, ADHD, conduct disorder, sleep disorders, and eating disorders) and includes substance use disorders and borderline personality disorder from the adult DIPS [26]. The Kinder-DIPS has good validity and reliability for Axis I disorders (child version, *κ* = 0.48–0.88) [27]. NSSI was assessed using the proposed *DSM-5 *criteria (proposed criteria in 2012). The proposed criteria as of 2012 and the final published version are comparable as follows. 

*Proposed diagnostic criteria for nonsuicidal self-injury (NSSI) for the fifth edition of the *Diagnostic and Statistical Manual of Mental Disorders (*DSM-5)* (As of November 2012, http://www.dsm5.org/) *used for the present study. *

- In the last year, the individual has, on 5 or more days, engaged in intentional self-inflicted damage to the surface of his or her body, of a sort likely to induce bleeding or bruising or pain (e.g., cutting, burning, stabbing, hitting, and excessive rubbing), for purposes not socially sanctioned (e.g., body piercing, tattooing, etc.), but performed with the expectation that the injury will lead to only minor or moderate physical harm. The behavior is not a common one, such as picking at a scab or nail biting.
- The intentional injury is associated with at least 2 of the following:
  1. psychological precipitant: interpersonal difficulties or negative feelings or thoughts, such as depression, anxiety, tension, anger, generalized distress, or self-criticism, occurring in the period immediately prior to the self-injurious act,
  2. urge: prior to engaging in the act, a period of preoccupation with the intended behavior that is difficult to resist,
  3. preoccupation: thinking about self-injury occurs frequently, even when it is not acted upon,
  4. contingent response: the activity is engaged in with the expectation that it will relieve an interpersonal difficulty, negative feeling, or cognitive state, or that it will induce a positive feeling state, during the act or shortly afterwards.
- The behavior or its consequences cause clinically significant distress or interference in interpersonal, academic, or other important areas of functioning. (This criterion is subject to final approval on the use of criteria that relate symptoms to impairment.)
- The behavior does not occur exclusively during states of psychosis, delirium, or intoxication. In individuals with a developmental disorder, the behavior is not part of a pattern of repetitive stereotypies. The behavior cannot be accounted for by another mental or medical disorder (i.e., psychotic disorder, pervasive developmental disorder, mental retardation, Lesch–Nyhan syndrome, stereotyped movement disorder with self-injury, or trichotillomania).
- The absence of suicidal intent has either been stated by the patient or can be inferred by repeated engagement in a behavior that the individual knows, or has learnt, is not likely to result in death.

Diagnostic criteria for NSSI according to *DSM-5* [9] are as follows:

- In the last year, the individual has, on 5 or more days, engaged in intentional self-inflicted damage to the surface of his or her body of a sort likely to induce bleeding, bruising, or pain (e.g., cutting, burning, stabbing, hitting, and excessive rubbing), with the expectation that the injury will lead to only minor or moderate physical harm (i.e., there is no suicidal intent).
- Note: The absence of suicidal intent has either been stated by the individual or can be inferred by the individual's repeated engagement in a behavior that the individual knows, or has learned, is not likely to result in death.
- The individual engages in the self-injurious behavior with one or more of the following expectations:
  1. to obtain relief from a negative feeling or cognitive state,
  2. to resolve an interpersonal difficulty,
  3. to induce a positive feeling state.
- Note: The desired relief or response is experienced during or shortly after the self-injury, and the individual may display patterns of behavior suggesting a dependence on repeatedly engaging in it.
- The intentional self-injury is associated with at least one of the following:
  1. interpersonal difficulties or negative feelings or thoughts, such as depression, anxiety, tension, anger, generalized distress, or self-criticism, occurring in the period immediately prior to the self-injurious act,
  2. prior to engaging in the act, a period of preoccupation with the intended behavior that is difficult to control,
  3. thinking about self-injury that occurs frequently, even when it is not acted upon.
- The behavior is not socially sanctioned (e.g., body piercing, tattooing, part of a religious or cultural ritual) and is not restricted to picking a scab or nail biting.
- The behavior or its consequences cause clinically significant distress or interference in interpersonal, academic, or other important areas of functioning.
- The behavior does not occur exclusively during psychotic episodes, delirium, substance intoxication, or substance withdrawal. In individuals with a neurodevelopmental disorder, the behavior is not part of a pattern of repetitive stereotypies. The behavior is not better explained by another mental disorder or medical condition (e.g., psychotic disorder, autism spectrum disorder, intellectual disability, Lesch-Nyhan syndrome, stereotyped movement disorder with self-injury, trichotillomania [hair pulling disorder], and excoriation [skin picking disorder]).

The criteria were reformulated as questions. Interrater reliability estimates for the diagnosis of NSSI were very good (*κ* = 0.90). Suicide attempts were also assessed at the end of the interview. Master's students in clinical child psychology were first systematically trained in conducting the interviews.

Participants were administered the Structured Clinical Interview for *DSM-IV* Axis II personality disorders [SCID-II; 29] to assess personality disorders. The SCID-II was found to be suitable for use among adolescents [28]. 

The *Global Assessment of Functioning *(GAF) [29] assesses overall patient functioning and symptom severity; these characteristics have been reliably associated with clinical diagnosis, psychopathologic symptoms, and other clinical outcome ratings [30, 31].

The *Questionnaire of Thoughts and Feelings* (QTF) is a self-report scale (37 items) designed to measure borderline-specific basic assumptions and negative feelings [32]. It is based on cognitive models and Linehan's biosocial model of BPD. The internal consistency within our sample was *α* = 0.97. 

The *Borderline Symptom List* (BSL-95) [33] is a self-rating instrument for specific assessment of borderline-typical symptomatology. The symptomatology is collected for the last week. The BSL-95 includes 95 items that are based on *DSM-IV *criteria, the revised version of the Diagnostic Interview for Borderline Personality Disorder, and the opinions of both clinical experts and borderline patients. It consists of seven subscales assessing self-perception, affect regulation, self-destruction, dysphoria, loneliness, intrusions, and hostility. Within our sample the internal consistency for the subscales ranged from *α* = 0.84 to 0.96. The internal consistency within the present sample for the total score was *α* = 0.98. 

The *Difficulties in Emotion Regulation Scale *(DERS) [34, 35] is a 36-item self-report questionnaire designed to assess multiple aspects of emotion dysregulation. The measure yields a total score and scores on six subscales (nonacceptance of emotional responses, difficulties engaging in goal-directed behavior, impulse control difficulties, lack of emotional awareness, limited access to emotion regulation strategies, and lack of emotional clarity). The internal consistency within the present sample was *α* = 0.96 for the total score, and for the subscales it ranged from *α* = 0.80 to 0.93.

The *Functional Assessment of Self-Mutilation* (FASM) [36, 37] is a self-report measure of the methods, frequency, and functions of NSSI. The internal consistency within our sample was *α* = 0.85 for the overall scale. 

The *Youth Self-Report* (YSR) [38, 39] measures a broad range of psychopathology. Internal consistency within the present sample was *α* = 0.96 for the total score, *α* = 0.94 for the internalizing score, and *α* = 0.90 for the externalizing score. 

The *Beck Depression Inventory-II *(BDI-II) [40]. The BDI-II consists of 21 items and assesses depressive symptoms in adolescents. The internal consistency within the present sample was *α* = 0.96.

The *Depression Anxiety Stress Scale* (DASS-21) [41, 42]. The DASS is a reliable and valid self-report questionnaire comprising three scales measuring depression, anxiety, and stress. The internal consistency within the present sample was *α* = 0.93 for the depression scale, 0.85 for the anxiety scale, 0.84 for the stress scale, and 0.94 for the total scale.

### 2.4. Data Analyses

Logistic regression analyses were conducted to evaluate group differences on diagnoses. Independent variables were the group levels, and the dependent variables the disorders. As we were interested in specific group differences, we set up orthogonal comparisons. The first comparison contrasted the nonclinical adolescent group (NCA) with the clinical groups (CCA, NSSI, NSSI-C). The second comparison contrasted the clinical control adolescents (CCA) with the two NSSI groups (NSSI and NSSI-C, adolescents with or without impairment/distress). The third comparison contrasted the two NSSI groups, that is, the NSSI and NSSI-C groups. Multivariate analyses of variance (MANOVAs) were used to compare the groups (NCA, CCA, NSSI-C, and NSSI) on dependent variables such as internalizing and borderline symptoms, which were arranged based on content-wise criteria. If the Levene test indicated that the variance homogeneity of an outcome was violated, we transformed it for the analysis (log 10 or sqrt). One-way between-groups analyses of variance (ANOVAs) and effect sizes (Cohen's *d*) were used to assess differences in externalizing psychopathology (YSR external), general psychopathology (YSR total), global functioning (GAF), and difficulties in emotion regulation (DERS). The same orthogonal contrasts as described above were used to analyse group differences. For the comparison of self-injurious behavior between the NSSI groups with and without impairment, two MANOVAs were conducted, for the severity of NSSI (frequencies, and number of methods) and functions of NSSI, respectively. Significance levels were set at *α* = 0.05. 

## 3. Results

### 3.1. Diagnostic Criteria of NSSI Disorder

The percentages of fulfilled B and C criteria for NSSI and the mean scores of frequency and strength of NSSI symptoms of adolescents with NSSI disorder and of adolescents with NSSI without impairment/distress are presented in Table 2. Data show that for the B criteria, psychological precipitant, frequent urges, and contingent responses were reported by at least 85% of the participants, whereas preoccupation with the behavior and difficulty resisting the urge were reported by less than 50% of the participants. For the C criteria, impairment at leisure time was reported most frequently, and distress was indicated by 69% of the adolescents with NSSI disorder. The highest endorsement (79%) was to the question regarding desire for help, which was added to better operationalize the impairment/distress criteria. This question was also answered affirmatively by 30% of adolescents who denied experiencing impairment or distress due to NSSI. 

### 3.2. Symptoms of NSSI

The frequencies of each methods of self-injury used by the adolescents with NSSI and NSSI-C are presented in Table 3. A group differentiation between minor and moderate/severe methods was not possible, as 94% of the NSSI group and 82% of the NSSI-C group engaged in minor and moderate/severe methods.  Table 4 shows the mean number of methods of NSSI performed, the experience of pain, the age of onset of NSSI, and received medical treatment. Further, group differences and effect sizes on severity and functions of NSSI are reported. There was no significant group effect for number of methods used, pain, and age of onset. Moreover, there was no significant group effect for the function of the NSSI behavior, *F* (4, 38) = 1.58, *p* = .20, but the automatic negative reinforcement, *F* (1, 41) = 4.73, *p* = .035, and positive reinforcement, *F* (1, 41) = 6.41, *p* = .015, were significantly more endorsed by the NSSI group compared with the NSSI-C group, which is also indicated by large effect sizes (Cohen's *d* = 1.08, 1.21). 

### 3.3. Diagnostic Correlates

Axis I and II diagnoses for the clinical samples are reported in Table 5. The mean number of diagnoses was 3.46 (SD = 1.80) for the NSSI group, 1.70 (SD = 1.2) for the CCA group, and 2.09 (SD = 0.70) for the NSSI-C group. According to our data, NSSI was comorbid with other psychopathological disorders in all but two subjects (5%). Major depression was the most frequent comorbidity, followed by social phobia and PTSD. Logistic regression analyses indicated that major depression was significantly more prevalent (OR = 5.78, *p* < .05) among the NSSI group compared with the CCA group. Table 5 shows odds ratios (ORs) and 95% confidence intervals for odds ratios for each diagnosis.

Adolescents with NSSI had relatively more diagnoses of PTSD and suicide attempts compared with the NSSI-C and CCA groups. In our sample, eight adolescents (20.5%) with NSSI fulfilled the criteria for BPD. Adolescents with NSSI but not fulfilling diagnostic criteria for BPD endorsed a mean of 2.3 (SD = 1.56, range 0–4) symptoms of BPD. Most frequent symptoms were, other than self-injurious behavior, affective instability and inappropriate, intense anger. Least frequent symptoms were identity disturbances and paranoid ideation/severe dissociative symptoms. 

### 3.4. Clinical Correlates

Table 6 shows results of one-way ANOVAs and MANOVAs. MANOVAs were performed for group comparisons of internalizing psychopathology (BDI-II, DASS subscales, and YSR internal) and symptoms of BPD (QTF and BSL-95). As expected, the NCA group showed the lowest scores of psychopathology. The NSSI group had significantly higher symptoms of depression (DASS and BDI) compared with the CCA group; there were no significant differences in anxiety symptoms. For the comparison of the QTF and BSL-95 scores, adolescents with BPD were excluded from adolescents with NSSI disorder. Between adolescents with NSSI disorder without BPD (QTF: Mdn = 3.24; BSL-95 : Mdn = 173.34) and adolescents with NSSI disorder and BPD (QTF : Mdn = 3.54; BSL-95: Mdn = 185.06) there was no significant difference, and effect sizes were small regarding the QTF total score (*U* = 59.50, *p* = .39, *r* = 0.17) and the BSL-95 total score (*U* = 37.00, *p* = .84, *r* = 0.05), but results have to be interpreted with caution as the sample size of adolescents with NSSI and BPD was very small (*n* = 8). 

The one-way ANOVAs yielded significant group differences for functional impairment (GAF), general psychopathology (YSR), externalizing symptoms (YSR external), and difficulties in emotion regulation (DERS) between nonclinical and clinical groups as well as between clinical controls and adolescents with NSSI. The differences between the NSSI and NSSI-C groups were statistically not significant but showed a trend toward higher psychopathology of the NSSI group. 

## 4. Discussion

We examined the proposed *DSM-5* criteria for an NSSI disorder in a female inpatient adolescent sample and investigated diagnostic and clinical correlates of NSSI, comparing adolescents with NSSI disorder, adolescents with NSSI without impairment/distress, adolescents with mental disorders without NSSI, and adolescents with no mental disorders. The results indicated that with the currently proposed *DSM-5* criteria for an NSSI disorder, a sample of adolescents could be identified who were more impaired than adolescents who were also hospitalized due to mental disorders but did not engage in NSSI. In addition, 80% of the adolescents with NSSI disorder did not meet criteria for BPD, supporting the evidence for a distinct diagnostic entity. 

For the proposed *DSM-5* diagnostic criteria for an NSSI disorder, in criteria B (intentional injury is associated with at least two of four symptoms) the highest frequency of agreement was for psychological precipitant, especially sadness and tension, and contingent response, especially relief from negative feelings. The lowest agreement was for preoccupation with the behavior. Results are in line with a community study [11], although they assessed criterion B1 (psychological precipitant) with two items of the FASM and we asked which feelings they experienced just before self-injuring. As in the Zetterqvist et al. [11] study, in our sample there were some (*n* = 12, 29% of adolescents of the NSSI group) who fulfilled the NSSI criteria A, B, D, and E but denied that the behavior caused them any impairment or distress. There is currently a general discussion on whether the impairment/distress criterion should be part of each diagnosis [43] and given the difficulty of objectively operationalizing impairment and distress [44]. Especially for patients with NSSI this might be a difficult question. These patients may see NSSI as a (temporary) solution to reduce distress [10, 11], and so they do not report impairment or distress. In an attempt to better operationalize the impairment/distress criterion, in the structured diagnostic interview Kinder-DIPS [45] there is an additional question: “Do you want help for this problem?” Whereas distress was reported by 69% of adolescents with NSSI disorder, a desire for help was affirmed by 80% and also by 30% of adolescents who denied having impairment or distress due to NSSI. When we compared the NSSI and NSSI-C groups, we found significantly less automatic positive and negative reinforcement as functions of NSSI in the NSSI-C group; furthermore, the NSSI-C group did not fulfil criteria for BPD, had fewer externalizing disorders, and, although not significant, showed a trend of reporting fewer depressive and borderline symptoms and less difficulties in emotion regulation. Future research using larger sample sizes should elaborate on this issue. 

The most common methods used for NSSI were cutting, carving, and scraping. This is in accordance with related literature [22, 46, 47]. The method “picked at a wound” should, as also suggested by others [11, 46], be excluded, as this method was also endorsed by 22% of adolescents in the nonclinical group. We were unable to differentiate between adolescents performing minor and moderate/severe NSSI methods due to a huge overlap. In the sample with NSSI disorder, the mean number of types of NSSI performed was 5.42, mean age of onset was 13 years, and 12% had received medical treatment. NSSI is mostly an impulsive behavior that 87% of the adolescents with NSSI disorder reported not thinking about at all or in the few minutes before engaging in NSSI. The most frequently reported functions were positive and negative automatic reinforcement, in line with [11]. 

As far as we know, this is the first study using clinical structured interviews and the suggested *DSM-5* criteria for NSSI to examine diagnostic correlates. Findings suggest that NSSI is comorbid with a wide range of diagnoses. The most common comorbid diagnoses were major depression, PTSD, and social phobia, supporting the results of others [13, 21] and a review by Nitkowski and Petermann [48]. Results are also in line with the chart review of inpatient adults with NSSI [15], characterized by high rates of internalizing disorders like depressive and anxiety disorders. All but one subject had at least one Axis I disorder in the Selby et al. [15] study; similarly, in our sample there were two adolescents with NSSI disorder without any comorbid diagnosis. The comorbidity with externalizing disorders would probably even be higher if the recruitment of this study would not focus on inpatient psychiatric adolescents as in Switzerland female adolescents with externalizing disorders are often placed in residential group homes with outpatient psychiatric and psychotherapeutic services. 

Our finding of a prevalence rate of 20% of adolescents with NSSI disorder also fulfilling diagnostic criteria for BPD corresponds to some studies [24, 48, 49] but is lower than the rate of 50% reported by Nock et al. [13]. On the criteria level, adolescents with NSSI disorder without a comorbid BPD endorsed a mean of 2.3 borderline symptoms compared with a mean of 0.3 endorsed by the clinical control adolescents. The least frequently endorsed criteria of the borderline symptoms were identity disturbances and paranoid/dissociative symptoms. Exploring different borderline features might be interesting, as a longitudinal study showed that behavioral impulsivity was an important symptom in explaining frequency of NSSI, low level of affective instability acted as a protective factor, and an unstable sense of self was less helpful in explaining the presence and initiation of NSSI among adolescents [50]. Dimensionally, adolescents with NSSI disorder were not significantly different from adolescents with BPD, although the scores of the adolescents with NSSI without BPD were lower, and for the BSL-95, below the clinical cut-off. Because self-injurious behavior is a criterion of BPD, there can be an association of NSSI and BPD; however, the current results indicate that NSSI disorder can be present without BPD. Nevertheless, future research has to investigate if adolescents with NSSI might develop additional BPD symptoms over time. Other than BPD, no other personality disorders were diagnosed in this sample. There may be a hesitancy to assign personality disorders in this age group [51]. 

In light of previous studies [11, 13, 21], a somewhat unexpected result was the low rate of alcohol and substance abuse or dependence. There was one adolescent with NSSI disorder fulfilling criteria for present substance abuse. On the interview on NSSI and in the FASM, three adolescents reported sometimes self-injuring under the influence of alcohol or drugs. One explanation of these results might be that the present sample was inpatient adolescents and therefore they did not have the opportunity to use drugs or alcohol on a regular basis. Furthermore, alcohol use in Switzerland is legal starting at age 16 (beer and wine) or 18 (all alcoholic beverages), respectively; that cultural differences might influence the results on an abuse in adolescents. However, as in other studies [11, 13], 54% of the NSSI group endorsed smoking regularly, compared with 10% of the CCA group. 

The majority (69%) of adolescents with NSSI disorder reported a suicide attempt, which is in line with the 70% found in the study by Nock et al. [13]. As all adolescents with NSSI disorder endorsed that they conducted NSSI without suicidal intent, NSSI has to be distinguished from suicidal behavior. This is also supported by the reports of some (18%) adolescents with NSSI disorder indicating that they engaged in NSSI to prevent a suicide attempt. Nevertheless, there is considerable overlap between NSSI and suicidal behavior. In two prospective studies, NSSI was shown to be a significant predictor for suicide attempts [18–20]. In our study, adolescents with NSSI disorder reported a mean age at onset of NSSI of 13 years, a mean age of 12 years for suicide ideations, and a mean age for the first suicide attempt of 14 years. This would be in line with Joiner's interpersonal theory of suicide [52] that attempting suicide requires both the desire and the capability to attempt suicide, and NSSI correlates with both. NSSI raises capability by allowing individuals to habituate to self-inflicted pain and violence [13] and it heightens risk for suicidal desire through association with emotional and interpersonal distress [18, 53]. Therefore, it is essential to identify why and how NSSI heightens the risk for suicide attempts. 

In addition to the diagnostic correlates, clinical correlates indicated that adolescents with NSSI disorder have, compared with adolescents with mental disorders without NSSI and in line with previous research, elevated rates of internalizing and externalizing symptoms [22, 25], low functioning [15], and difficulties in emotion regulation [23]. These findings complement the picture of highly impaired adolescents with NSSI disorder. 

Several limitations of this study should be noted. Our sample consisted of female adolescents admitted to a psychiatric unit and thus may not generalize to other samples. Second, our data were cross-sectional. Third, our subsample sizes were small, so the power was limited for some analyses. Fourth, even though NSSI will be a disorder in Section 3 of the *DSM-5* [9], the proposed criteria are not finalized.

Strengths of the study were the use of the proposed *DSM-5* diagnostic criteria for NSSI, tackling the problems of previous research on self-injury, where different definitions were used, and investigating samples with repetitive and single episodes of NSSI. Another strength is the use of a multimethod assessment, employing self-report measures and structured clinical interviews. 

Implications of these results are that a precise and comprehensive diagnostic assessment including NSSI should be conducted routinely. On one side, NSSI is a highly impairing disorder on its own for the patients themselves, relatives, and friends, and on the other side, it is also a risk factor for suicidal behavior. In summary, our study suggests that the proposed *DSM-5* criteria for NSSI are useful and necessary to promote research on aetiology, course, and the development of effective treatment strategies and interventions for adolescents suffering from NSSI.

## Figures and Tables

**Table 1:** Demographic and psychosocial characteristics of adolescents with NSSI disorder (NSSI), compared with non-clinical adolescents (NCA), clinical controls without NSSI (CCA), and adolescents with NSSI without impairment/distress (NSSI-C).

Characteristic	NCA (*n* = 37)	CCA (*n* = 20)	NSSI-C (*n* = 11)	NSSI disorder (*n* = 39)	Welch's *F* (3,33.36)
Mean age (SD) in years	14.60 (1.02)	15.93 (1.52)	17.08 (1.92)	15.94 (1.42)	12.19**F (3,79)
Mean no. of school years (SD)	8.40 (1.08)	9.25 (1.58)	9.33 (1.41)	9.16 (1.10)	2.88*
Number (percentage) living with parents^a^	31 (100)	15 (93.8)^a^	11 (100)	26 (83.9)^b^	*χ* ^2^ (9) = 10.2
Number (percentage) whose parents have joint custody^c^	31 (86.1)	12 (75.0)	7 (70.0)	20 (66.7)	*χ* ^2^ (9) = 8.04

Note. *p < .05, **p < .01. ^a^One was in another child and adolescent psychiatric clinic ^b^three children lived in a supervised residential group, one in a foster family, and one in another child and adolescent psychiatric clinic, ^c^the rest had mothers with sole custody.

**Table 2:** Frequency and percentage of the proposed B and C diagnostic criteria for NSSI for the DSM-5, of adolescents with NSSI (NSSI disorder) and adolescents with NSSI without impairment/distress (NSSI-C).

Proposed criterion	NSSI disorder (*n* = 39) No. (%)	NSSI disorder frequency^a^ M (SD)	NSSI disorder strength^b^ M (SD)	NSSI-C (*n* = 10) No. (%)
B1: Psychological precipitant	38 (97.4)			10 (100)
Sadness	30 (76.9)	2.29 (0.98)	2.38 (0.95)	7 (70.0)
Tension	29 (74.4)	1.82 (1.02)	1.89 (1.09)	5 (50.0)
Anger	24 (61.5)	1.68 (1.14)	1.69 (1.19)	6 (60.0)
Distress	23 (59.0)	1.66 (1.19)	1.70 (1.18)	5 (50.0)
Self-criticism	19 (48.7)	1.38 (1.18)	1.50 (1.23)	6 (60.0)
Anxiety	8 (20.5)	0.76 (1.13)	0.83 (1.12)	2 (20.0)
B2:				
Preoccupation with behaviour	18 (46.2)			7 (70.0)
Difficulties resisting the urge	15 (38.5)	2.47 (0.80)		4 (40.0)
B3: Urge occurs frequently	35 (89.7)	2.42 (0.72)	2.44 (0.64)	5 (50.0)
B4:				
Contingent response	34 (87.2)			7 (70.0)
Relief from negative feelings				
Before	10 (25.6)	0.63 (1.00)		4 (40.0)
During	14 (35.9)	1.00 (1.19)		3 (30.0)
After	21 (53.8)	1.66 (1.24)		7 (70.0)
Fewer interpersonal problems				
Before	2 (5.1)	0.15 (0.59)		2 (20.0)
During	5 (12.8)	0.35 (0.86)		2 (20.0)
After	4 (10.3)	0.34 (0.82)		3 (30.0)
Feel better				
Before	7 (17.9)	0.47 (0.98)		4 (40.0)
During	9 (23.1)	0.68 (1.12)		2 (20.0)
After	18 (46.2)	1.32 (1.32)		6 (50.0)
Reward				
Before	1 (2.6)	0.11 (0.52)		0 (0)
During	1 (2.6)	0.08 (0.50)		0 (0)
After	4 (10.3)	0.27 (0.80)		0 (0)
Preventing suicide attempt				
Before	7 (17.9)	0.94 (1.08)		1 (10.0)
During	6 (15.4)	0.38 (0.87)		2 (20.0)
After	3 (7.7)	0.28 (0.77)		0 (0)
C: Distress, Impairment				
Impairment	39 (100)	1.97 (0.77)		0 (0)
Home	9 (23.1)	0.94 (0.95)		0 (0)
School	8 (20.5)	0.94 (0.93)		0 (0)
Leisure time	13 (33.3)	1.12 (1.02)		0 (0)
Friends	10 (25.6)	0.88 (.99)		0 (0)
Distress	27 (69.2)			0 (0)
Want help:	31 (79.5)			3 (30)

Note. ^a^Frequency scale 0–3 (0 = never, 1 = sometimes, 2 = often, 3 = very often) ^b^Strength scale 0–3 (0 = not at all, 1 = a little, 2 = strong, 3 = very strong).

**Table 3:** Frequency of methods of self-injurious behaviour assessed by the FASM in adolescents with NSSI (NSSI disorder) and adolescents with NSSI without impairment/distress (NSSI-C).

Method	NSSI disorder (*n* = 33)	NSSI-C (*n* = 11)
	No. (%)	No. (%)
Moderate/severe NSSI		
Cutting/carving on skin	32 (97.0)	9 (81.8)
Scraping	21 (63.6)	8 (72.7)
Burning skin	13 (39.4)	5 (45.5)
Rubbing skin to draw blood	9 (27.3)	3 (27.3)
Self-tattooing	3 (9.1)	1 (9.1)
Total moderate/severe methods	33 (100)	10 (90.9)

Minor NSSI		
Picking at a wound	24 (72.7)	7 (63.6)
Biting self	23 (69.7)	3 (27.3)
Hitting self	19 (57.6)	6 (54.4)
Inserting objects under skin or nails	9 (27.3)	0 (0)
Pulling out one's own hair	6 (18.2)	0 (0)
Picking areas of the body to the point of drawing blood	6 (18.2)	0 (0)
Total minor methods	31 (93.3)	10 (90.9)

Note. FASM: Functional Assessment of Self-Mutilation.

**Table 4:** Means, standard deviations (SDs), and effect sizes (Cohen's *d*) of the FASM, in adolescents with NSSI (NSSI disorder) and adolescents with NSSI without impairment/distress (NSSI-C).

FASM item	NSSI disorder (*n* = 33) M (SD)	NSSI-C (*n* = 12) M (SD)	*F*(1,41)	Cohen's *d*
No. of methods used	5.42 (2.18)	4.12 (2.00)	3.03	0.62
Pain^a^	3.18 (0.98)	2.91 (0.70)	0.72	0.32
Medical treatment by medical staff	No. 4 (12.1%)	No. 1 (8.3%)	*χ* ^2^ = 0.11	
Age of onset (years)	13.05 (1.73)	13.00 (2.41)	0.01	0.02
Function			*F*(4,38) = 1.58	
Automatic negative reinforcement	2.43 (0.84)	1.54 (0.81)	4.73*	1.08
Automatic positive reinforcement	2.08 (0.71)	1.33 (0.51)	6.41*	1.21
Social negative reinforcement	0.42 (0.48)	0.27 (0.34)	0.95	0.36
Social positive reinforcement	0.58 (0.37)	0.64 (0.58)	0.20	0.06

Note. ^a^On a scale from 4 (no pain) to 1 (severe pain); *p < .05.

**Table 5:** Diagnostic correlates of adolescents with clinical diagnoses without NSSI (CCA), adolescents with NSSI without impairment/distress (NSSI-C), and adolescents with NSSI (NSSI), as well as logistic regressions and orthogonal comparisons between clinical controls and NSSI (CCA versus NSSI) and between NSSI disorder and NSSI-C (NSSI versus NSSI-C).

	CCA (*n* = 20) *N* (%)	NSSI-C (*n* = 11) *N* (%)	NSSI (*n* = 39) *N* (%)	CCA versus NSSI exp⁡(*b*) = OR [95% CI]	NSSI versus NSSI-C exp⁡(*b*) = OR [95% CI]
Major depression	6 (30)	8 (72.7)	31 (79.5)	5.78 [1.12–29.85]*	1.36 [0.29–6.34]
Social phobia	5 (25)	3 (27.3)	15 (38.5)	1.05 [0.20–5.60]	1.82 [0.41–8.00]
PTSD	1 (5)	2 (18.2)	11 (28.2)	4.00 [0.32–50.23]	1.90 [0.35–10.28]
BPD	0	0	8 (20.5)	NA	NA
Specific phobia	4 (20)	1 (9.1)	7 (17.9)	0.53 [0.05–5.86]	2.33 [0.26–21.36]
ODD	2 (10)	1 (9.1)	5 (12.8)	0.85 [0.07–10.61]	1.56 [0.16–15.00]
Bulimia Nervosa	0	0	5 (12.8)	NA	NA
Dysthymia	2 (10)	2 (18.2)	4 (10.3)	1.89 [0.23–15.74]	0.55 [0.09–3.47]
Conduct disorder	0	0	4 (10.3)	NA	NA
OCD	4 (20)	0	2 (5.1)	NA	NA
Agoraphobia	2 (10)	1 (9.1)	2 (5.1)	1.80 [0.10–31.99]	0.57 [0.05–6.97]
ADHD	0	0	2 (5.1)	NA	NA
Anorexia nervosa	3 (15)	2 (18.2)	1 (2.6)	1.19 [0.17–8.47]	0.13 [0.01–1.54]
Panic disorder	1 (5)	0	1 (2.6)	NA	NA
GAD	2 (10)	0	1 (2.6)	NA	NA
Suicide attempts	4 (20)	6 (54.4)	27 (69.2)	4.50 [0.89–22.74]	1.88 [0.48–7.36]
Smoking	2 (10)	3 (27.3)	21 (53.8)	3.38 [0.47–24.29]	3.11 [0.72–13.51]

	M (SD)	M (SD)	M (SD)	*T*(67)	*T*(67)

No. of diagnoses	1.70 (1.22)	2.09 (0.70)	3.46 (1.80)	2.50**	2.62, *p* = .07 *d* = 1.0

Note. *p < .05,  **p < .01. ADHD: Attention deficit hyperactivity disorder, ODD: Oppositional Deviant Disorder, GAD: Generalized Anxiety Disorder, BPD: Borderline Personality Disorder, OCD: Obsessive Compulsive Disorder, PTSD: Posttraumatic Stress Disorder, NA: not applicable.

**Table 6:** Clinical correlates of non-clinical adolescents (NCA), clinical controls (CCA), adolescents with NSSI without impairment/distress (NSSI-C), and adolescents with NSSI disorder (NSSI), as well as MANOVA and ANOVA with orthogonal contrasts and effect sizes (Cohen's *d*) between non-clinical and clinical groups (NCA versus rest), clinical controls and NSSI (CCA versus NSSI total), and NSSI disorder versus NSSI-C.

Questionnaire	NCA M (SD)	CCA M (SD)	NSSI-C M (SD)	NSSI M (SD)	NCA versus rest	Cohen's *d*	CCA versus NSSI total	Cohen's *d*	NSSI disorder versus NSSI-C	Cohen's *d*
ANOVA					T (91.52)		T (40.22)		T (38.67)	
GAF	94.20 (3.87)	59.55 (6.40)	56.27 (4.50)	53.70 (10.17)	34.84**	7.26	−2.55*	0.80	1.19	0.38

					T (92)		T (92)		T (92)	
YSR Total^a^	51.60 (14.23)	83.79 (19.13)	98.93 (18.11)	111.31 (26.77)	11.72**	2.44	2.97**	0.62	1.22	0.25
YSR. EXT^a^	8.08 (4.32)	12.91 (1.74)	18.28 (9.38)	21.31 (11.32)	5.89**	1.23	2.17*	0.45	0.48	0.10

DERS	70.59 (16.89)	97.79 (24.14)	108.16 (17.55)	123.42 (25.80)	8.25**	1.72	2.76**	0.58	2.09	0.44

MANOVA 1					*F* (3,92)		*F* (3,92)		*F* (3,92)	
DASS depression^b^	1.25 (1.76)	8.84 (5.73)	11.83 (6.37)	13.82 (4.56)	165.85**	2.88	7.86**	0.81	1.51	0.41
DASS anxiety^b^	1.84 (2.19)	7.63 (4.49)	7.56 (4.71)	8.95 (5.26)	68.08**	1.89	0.11	0.17	0.80	0.28
DASS stress^b^	3.68 (2.74)	8.83 (4.40)	10.60 (4.37)	11.38 (4.60)	71.73**	1.88	2.71	0.54	0.20	0.16
BDI^b^	5.57 (5.87)	23.36 (13.11)	30.22 (9.38)	36.32 (12.32)	155.19**	2.70	11.07**	1.03	3.16	0.74
YSR. INT^b^	8.18 (6.58)	25.28 (9.67)	31.37 (8.29)	33.75 (10.04)	169.24**	2.78	6.47*	0.91	0.51	0.29

MANOVA 2					*F* (3,72)		*F* (3,72)		*F* (3,72)	
QTF^ac^	1.44 (0.36)	2.33 (0.90)	2.99 (0.48)	3.21 (0.83)	100.00**	2.42	12.91**	0.94	0.25	0.42
BSL-95^ac^	38.04 (17.47)	120.47 (76.01)	140.80 (64.29)	186.62 (64.93)	108.38**	2.45	4.79*	0.75	1.69	0.62

Note. GAF: Global Assessment of Functioning; YSR: Youth Self Report, INT: internal, EXT: external; DASS: Depression Anxiety Stress Scales; BDI: Beck's Depression Inventory; QTF: Questionnaire of Thoughts and Feelings; BSL-95: Borderline Symptom List; DERS: Difficulties in Emotion Regulation Scale; *p < .05; **p < .01; ***p < .001. ^a^log transformed/^b^SQRT transformed, ^c^adolescents with BPD were excluded from these analyses.
